# Indocyanine green angiography findings in patients with nonfamilial amyloidosis

**DOI:** 10.1007/s12348-012-0085-7

**Published:** 2012-05-24

**Authors:** Sonia Attia, Rim Kahloun, Sameh Mbarek, Olfa Harazallah, Habib Skhiri, Salim Ben Yahia, Moncef Khairallah

**Affiliations:** 1 Department of Ophthalmology, Fattouma Bourguiba University Hospital, Faculty of Medicine and University of Monastir, 5019 Monastir, Tunisia; 2 Department of Internal Medicine, Fattouma Bourguiba University Hospital, Faculty of Medicine and University of Monastir, 5019 Monastir, Tunisia; 3 Department of Nephrology, Faculty of Medicine and University of Monastir, Fattouma Bourguiba University Hospital, Faculty of Medicine and University of Monastir, 5019 Monastir, Tunisia

**Keywords:** Choroidopathy, Indocyanine green angiography, Nonfamilial amyloidosis

## Abstract

**Purpose:**

The purpose of this study is to assess indocyanine green angiographic findings in patients with nonfamilial amyloidosis.

**Methods:**

The method used was a prospective study including seven patients (14 eyes) with nonfamilial amyloidosis. All patients underwent detailed ophthalmic clinical examination, fundus photography, and indocyanine green angiography (ICGA). Fluorescein angiography (FA) was performed in four patients.

**Results:**

Of the seven patients, four (57.1 %) were male. Mean age was 49.5 years. Six patients had renal amyloidosis and one patient had systemic amyloidosis. Mean best-corrected visual acuity was 20/25. Fundus and FA findings included cotton-wool spots (28.5 %), retinal hemorrhages (14.3 %), retinal pigment epithelial changes (21.4 %), serous retinal detachment (7.1 %), optic disk edema or staining (7.1 %), area of peripheral retinal capillary non-perfusion (7.1 %), disseminated peripheral punctiform hyperfluorescence (21.4 %), and subretinal pooling (7.1 %). Fundus examination results were unremarkable in eight eyes (57.1 %). ICGA showed abnormal findings in all eyes. These included diffuse or focal/multifocal choroidal vascular staining appearing at the late phase and prevailing in peripheral fundus (100 %), hyperfluorescent fleecy lesions appearing at the late phase and also prevailing in peripheral fundus (28.5 %), hypofluoresent areas of variable sizes (85.7 %), and pinpoints (71.4 %).

**Conclusions:**

Our results show that a subclinical, fairly typical choroidal involvement, detectable only by ICGA, is common in patients with nonfamilial amyloidosis. ICGA may be useful in better understanding the pathogenesis of amyloidosis choroidopathy and in establishing a diagnosis of amyloidosis in atypical or incomplete clinical presentations.

## Introduction

Amyloidosis is a systemic disorder, characterized by an aberrant deposition, in single or multiple organs, of insoluble chains of polypeptides derived from a portion of light chain of immunoglobulins [1]. It may affect one (localized) or multiple organs (systemic) and can have a family pedigree (familial). The underlying cause may be unknown (primary), although amyloid can develop as a consequence of chronic inflammatory or neoplastic conditions (secondary amyloidosis) [1, 2].

Amyloid deposition has been reported in ocular adnexae, orbit, and components of the globe such cornea, iris, trabecular meshwork, sclera and vitreous body [2–4]. Choroidal involvement in patients with nonfamilial amyloidosis (NFA) has been rarely described [5, 6].

The purpose of the present study was to assess prospectively indocyanine green angiography (ICGA) features in patients with NFA.

## Methods

The study included seven consecutive patients (14 eyes) diagnosed with NFA and examined at Fattouma Bourguiba University Hospital (Monastir, Tunisia). The diagnosis of amyloidosis was based on kidney biopsy in five patients, on colonic biopsy in one patient, and on labial biopsy in one patient.

All patients underwent detailed ophthalmic evaluation including measurement of Snellen best-corrected visual acuity (BCVA), slit-lamp examination, tonometry, and dilated fundus examination with non contact and contact lenses. Fundus photography and ICGA were performed in all patients. Fluorescein angiography (FA) was performed in four patients.

The complete protocol was reviewed and approved by the ethics and research committees of our institution, and all patients provided informed consent.

## Results

Of the seven patients, four (57.1 %) were male and three (42.9 %) were female. The patients’ ages ranged from 36 years to 70 years (mean, 49.5 years). The demographic characteristics and systemic manifestations are listed in Table 1. Duration of the disease ranged from few months to 15 years. Five patients had localized renal amyloidosis and two patients had systemic amyloidosis with renal and colonic involvement for the first one (patient 5) and cardiac and hepatic involvement for the second one (patient 7). Amyloidosis was primary in 6 patients and secondary to tuberculosis in 1 patient. Two patients (28.5 %) had systemic hypertension and 3 patients (42.8 %) were hemodialysed for advanced kidney failure.

**Table 1:** Demographic and clinical characteristics of patients

Patient no	Age (years)	Sex	Type of amyloidosis	Duration of the disease (years)	Systemic hypertension	Hemodialysis
1	43	M	Primary/localized	4	−	−
2	70	F	Primary/localized	3	+	+
3	65	M	Secondary/localized	1	+	−
4	36	F	Primary/localized	1	−	+
5	37	M	Primary/systemic	15	−	−
6	47	F	Primary/localized	5	−	+
7	49	M	Primary/systemic	<1	−	−

*M* male, *F* female

No patient had ocular complains except one patient (patient 7) who experienced a decrease in visual acuity in his right eye few days before examination. BCVA ranged from light perception to 20/20 (mean, 20/25).

**Fig. 1:**
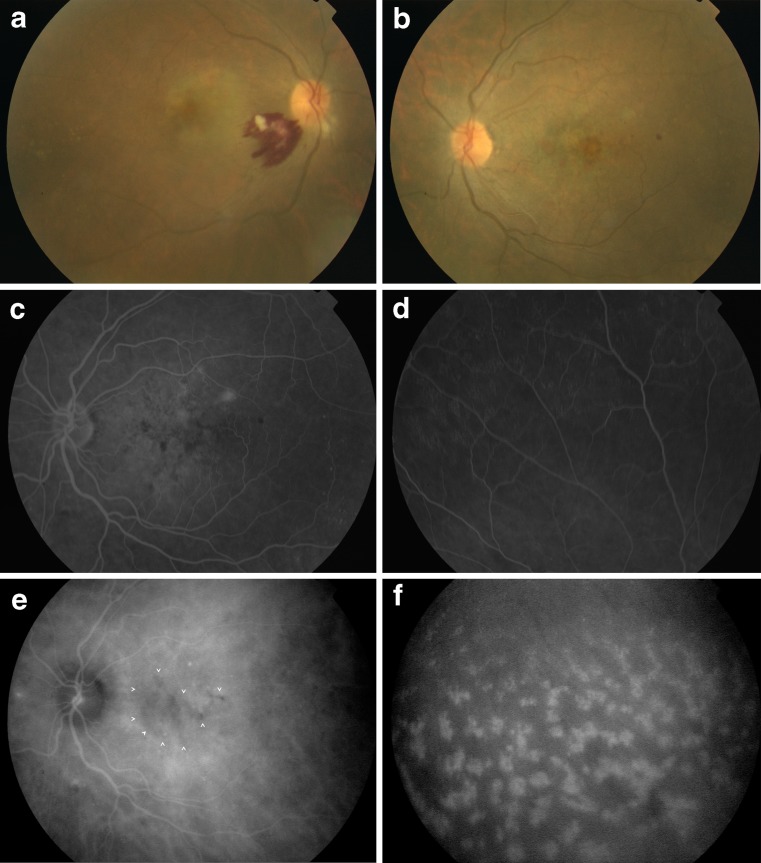
Patient 1. **a** Fundus photograph of the right eye shows retinal hemorrhages, cotton-wool spots, and retinal pigment epithelial changes in the macular area. **b** Fundus photograph of the left eye shows retinal pigment epithelial changes. Mid-phase fluorescein angiogram of the left eye shows retinal pigment epithelium changes, retinal vascular leakage (**c**), and disseminated peripheral punctiform hyperfluorescence (**d**). **e** Mid-phase indocyanine green angiogram of the left eye showing areas of macular hypofluorescence (*white arrow heads*). **f** Late-phase indocyanine green angiogram of the same eye shows hyperfluorescent fleecy lesions in the midperiphery

**Fig. 2:**
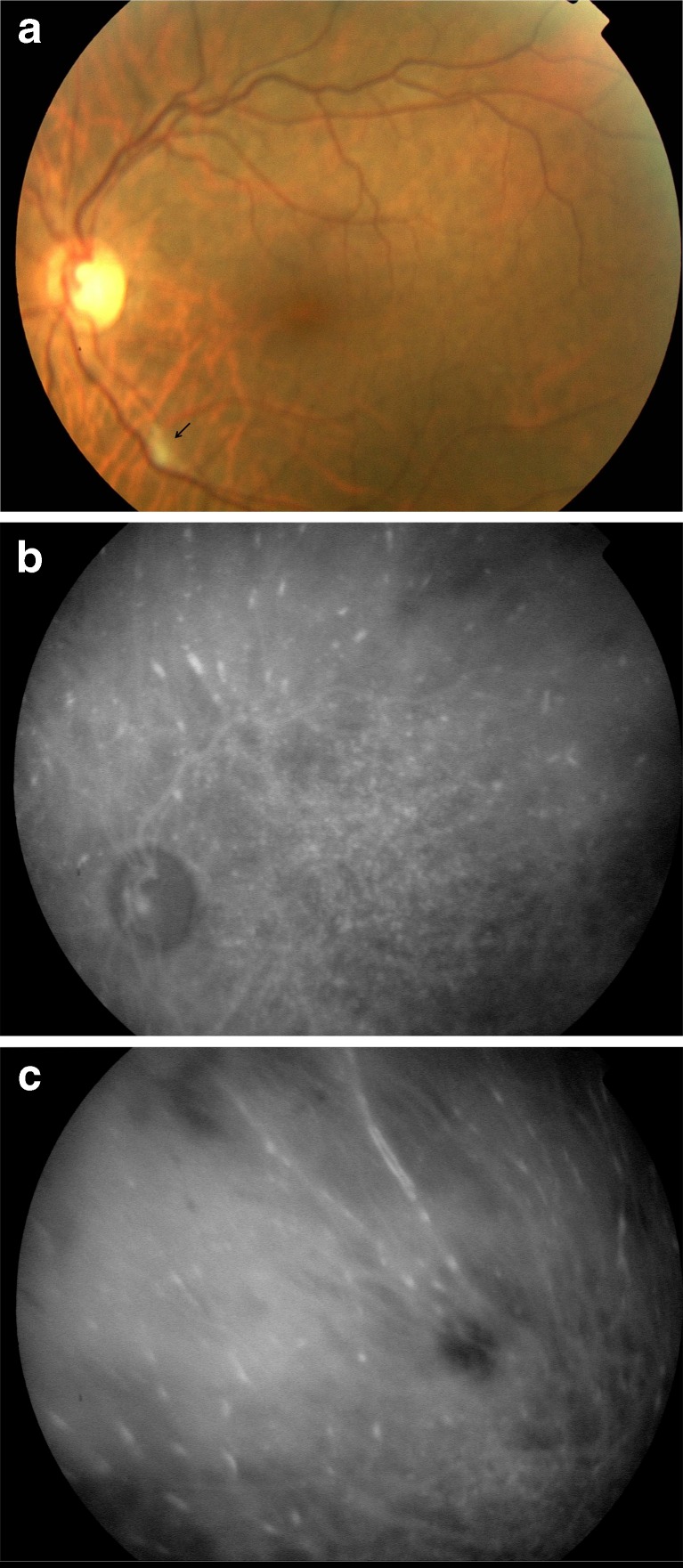
Patient 4. **a** Fundus photograph of the left eye shows a cotton-wool spot (*black arrow*). Late-phase indocyanine green angiogram shows pinpoints with multifocal choroidal vascular staining (**b**) and hyperfluorescent streaks that closely follow the course of choroidal vessels in the midperiphery (**c**)

Fundus findings included cotton-wool spots (4 eyes; 28.5 %; Figs. 1a and 2a), retinal hemorrhages (2 eyes; 14.3 %; Fig. 1a), retinal pigment epithelial changes (3 eyes; 21.4 %; Figs. 1a and b), serous retinal detachment (1 eye; 7.1 %), and optic disc edema (1 eye; 7.1 %). Fundus examination was normal in eight eyes (57.1 %). No cases of conjunctival involvement or vitreous opacities were recorded in our patients.

FA revealed retinal pigment epithelial changes (3 eyes; 21.4 %; Fig. 1c), optic disc staining (1 eye; 7.1 %), subretinal pooling (1 eye; 7.1 %), limited area of peripheral retinal capillary non-perfusion (1 eye; 7.1 %), and disseminated peripheral punctiform hyperfluorescence (3 eyes, 21.4 %; Fig. 1d).

ICGA showed choroidal involvement in all eyes (Table 2). Diffuse choroidal vascular staining was observed in all eyes. It presented in the form of hyperfluorescent streaks that became visible at the late phase, predominating in peripheral fundus and appearing to closely follow the course of choroidal vessels (Fig. 2c). Focal/multifocal choroidal vascular staining were observed in six eyes (42.8 %; Fig. 2b).

**Table 2:** Funduscopic, fluorescein, and ICG angiographic findings in patients with nonfamilial amyloidosis

Patient no	Funduscopic findings	Fluorescein angiographic findings	ICG angiographic findings
1	Cotton-wool spots OU; retinal hemorrhages OU; RPE changes OU	RPE changes OU; disseminated peripheral punctiform hyperfluorescence OU	Diffuse choroidal vascular staining OU; hyperfluorescent fleecy lesions OU; pinpoints OU; areas of hypofluorescence OU
2	None	Not done	Diffuse choroidal vascular staining OU; focal/multifocal choroidal vascular staining OD; areas of hypofluorescence OU
3	None	Area of peripheral retinal capillary non-perfusion	Diffuse choroidal vascular staining OU; pinpoints OU; areas of hypofluorescence OU
4	Cotton-wool spots OS	Not done	Diffuse choroidal vascular staining OU; focal/multifocal choroidal vascular staining OS; pinpoints OU; areas of hypofluoresence OU
5	RPE changes OD	RPE changes OD	Diffuse choroidal vascular staining OU; focal/multifocal choroidal vascular staining OU; pinpoints OU; areas of hypofluorescence OU
6	None	Not done	Diffuse choroidal vascular staining OU; focal/multifocal choroidal vascular staining OS
7	Cotton-wool spots OD; optic disc staining OD; serous retinal detachment OD	Optic disc edema OD; disseminated peripheral punctiform hyperfluorescence OD; subretinal pooling OD	Diffuse choroidal vascular staining OU; focal/multifocal choroidal vascular staining OS; hyperfluorescent fleecy lesions OU; pinpoints OU

*ICG* indocyanine green, *RPE* retinal pigment epithelial, *OD* right eye, *OS* left eye, *OU* both eyes

Other ICGA findings included hyperfluorescent fleecy lesions appearing at the late phase and also predominating in peripheral retina in 4 eyes (28.5 %; Fig. 1f), multiple punctuate hyperfluorescences or pinpoints in 10 eyes (71.4 %; Fig. 2b), and hypofluoresent areas of variable sizes in 12 eyes (85.7 %; Fig. 1e).

## Discussion

Ocular involvement in amyloidosis is uncommon including corneal opacities, secondary glaucoma, pupillary disturbances, recurrent subconjunctival hemorrhages, recurrent periocular subcutaneous hemorrhages, conjunctival infiltration, sicca syndrome, ptosis, proptosis or globe displacement, periocular mass or tissue infiltration, limitations in ocular motility and diplopia, vitreous deposits, retinal hemorrhages, cotton-wool spots, pinpoint white amyloid opacities over the retinal surface, sheathing of retinal vessels, and retinal vascular closure [2, 7–12].

Choroidal involvement in NFA has been rarely described in literature, and reported findings include hypofluorescent areas with hypofluorescent lines in the midperiphery, hyperfluorescent streaks in the peripapillary area [5], and diffuse occlusion of the choriocapillaris [6].

To the best of our knowledge, our series is the largest and the first to characterize and analyze prospectively ICGA findings in patients with NFA. Our data show that a subclinical choroidal involvement is common in amyloidosic patients. Diffuse or focal/multifocal choroidal vascular staining appearing at the late phase and predominating in peripheral fundus was the main finding, occurring in all eyes. Similar findings were previously described in few patients with familial amyloidotic polyneuropathy [11, 12]. Other common findings in our series included hyperfluorescent fleecy lesions, appearing at the late phase and predominating in peripheral fundus, and pinpoints.

Diffuse or focal/multifocal choroidal vascular staining might correspond to hyperfixation of indocyanine green (ICG) dye by deposits of amyloid substance in choroidal vessels. As well, pinpoints and fleecy lesions could correspond to hyperfixation of ICG dye by deposits of amyloid substance in the choriocapillaris stroma and retinal pigment epithelium. In fact, histopathological studies confirmed that amyloid substance is synthesized by retinal pigment epithelium with deposition of amyloid seeping through retinal vessels into vitreous and in the choriocapillaris [11, 13].

Hypofluoresent areas of variable sizes were encountered in over 80 % of our patients. They might be caused by choroidal vascular occlusion that could result from the amyloidosis itself, the subsequent nephritis, observed in six patients, and/or systemic hypertension, observed in two patients.

No case of vitreous opacity was recorded in our series. Indeed, amyloid deposition in the vitreous is usually associated with the hereditary amyloidoses rather than NFA [2, 3].

In conclusion, amyloidosis choroidopathy may present with a wide spectrum of subclinical ICGA findings. ICGA may be useful in assessing and quantifying the extent of amyloidosis choroidopathy and in better understanding its pathogenesis. It may also help establish diagnosis in challenging cases of amyloidosis. Additional studies are required to clarify further the role of ICGA in diagnosing amyloidosis and evaluating its severity.
